# Leveraging annotation-based modeling with Jump

**DOI:** 10.1007/s10270-016-0528-y

**Published:** 2016-05-07

**Authors:** Alexander Bergmayr, Michael Grossniklaus, Manuel Wimmer, Gerti Kappel

**Affiliations:** 1 TU Wien, Favoritenstrasse 9-11, 1040 Vienna, Austria; 2 University of Konstanz, P.O. Box 188, 78457 Konstanz, Germany

**Keywords:** Java annotations, UML profiles, Model-based software engineering, Forward engineering, Reverse engineering

## Abstract

The capability of UML profiles to serve as annotation mechanism has been recognized in both research and industry. Today’s modeling tools offer profiles specific to platforms, such as Java, as they facilitate model-based engineering approaches. However, considering the large number of possible annotations in Java, manually developing the corresponding profiles would only be achievable by huge development and maintenance efforts. Thus, leveraging *annotation-based modeling* requires an automated approach capable of generating platform-specific profiles from Java libraries. To address this challenge, we present the fully automated transformation chain realized by Jump, thereby continuing existing mapping efforts between Java and UML by emphasizing on annotations and profiles. The evaluation of Jump shows that it scales for large Java libraries and generates profiles of equal or even improved quality compared to profiles currently used in practice. Furthermore, we demonstrate the practical value of Jump by contributing profiles that facilitate reverse engineering and forward engineering processes for the Java platform by applying it to a modernization scenario.

## Introduction

Since the introduction of the profile mechanism in UML, numerous profiles have been developed [65], many of which are available by the OMG standardization body [60]. Also in industry, their practical value has been recognized as today’s modeling tools offer already predefined stereotypes covered by profiles. They are considered as a major ingredient for current model-based software engineering approaches [10] by providing features supplementary to the UML standard metamodel. This powerful capability of profiles can also be exploited in terms of an annotation mechanism [71]. As a result, they leverage *annotation-based modeling*, where defined stereotypes show similar capabilities as annotations over program elements [24, 56].

Annotating program elements is widely adopted in practice [66, 69], and various programming languages provide concepts to support them, e.g., annotations in Java and Scala, attributes in C#, and decorators in Python. For the scope of this article, we focus on Java annotations since they have already been introduced in [63], and therefore, many well-known libraries embrace them. For instance, the Java Persistence API (JPA) [42] provides annotations to denote strong and weak entities, Enterprise Java Beans (EJB)^1^ [25] defines annotations to manage the state of session beans, and recently the Checker framework [17] uses type annotations introduced in Java 8 to indicate that an expression is never null. Hence, deriving stereotypes from established programming libraries to produce corresponding UML profiles on the model level is desirable [4, 31, 49]. For instance, IBM’s Rational Software Architect provides profiles for certain Java libraries. By applying such profiles, platform-independent models (PIMs) are refined into models specific to a platform (PSMs), where the platform refers to the library from which the profile was derived. Turning this forward engineering (FE) perspective into a reverse engineering (RE) one, existing programs can be represented as UML models that capture annotations by applying the corresponding profiles. Therefore, platform-specific profiles and their application are beneficial from both perspectives. In a RE process, model analyzers can exploit captured stereotypes to facilitate comprehension [15], whereas profiled UML models, i.e., models to which profiles are applied, pave the way for model transformers to generate richer program code in an FE process [71].

For that reason, we have realized Jump [6] that enables UML profiles to be generated automatically from Java libraries, which use annotations. Considering the large number of possible annotations in Java, manually developing the corresponding profiles would only be achievable by a huge development and maintenance effort. For instance, in the ARTIST project [5], we are confronted with this problem, as we work toward a model-based engineering approach for modernizing applications by novel cloud offerings. This involves representing PSMs that refer to the platform of existing applications, e.g., the JPA, when considering persistence, and the platform of “cloudified” applications, e.g., the Objectify library [57], when considering cloud datastores. Supporting JPA annotations on the model level facilitates distinguishing between plain association and composition relationships and precisely deciding on multiplicities, which, in general, is not easy to grasp [11]. UML models profiled by Objectify annotations enable generating method bodies even from a structural viewpoint. These examples highlight the practical value of platform-specific RE and FE tools, which are developed in the ARTIST project.

In this article, we present the fully automatic transformation chain realized by Jump. We propose an effective conceptual mapping between the two considered technical spaces [41, 48]: Java and UML. Our approach targets users who employ UML in order to realize reverse engineering and forward engineering processes where software artifacts are implemented in Java. Therefore, we continue the long tradition of investigating mappings between Java and UML [26, 36, 46, 54]. However, in this article, we also address Java annotations and UML profiles in the mapping process. This necessitates overcoming existing heterogeneities that, e.g., refer to the target specification of Java annotations and other peculiarities of how Java annotation types are declared. In this respect, we discuss the support of current modeling tools to represent Java annotations in UML and highlight the benefits of the mapping realized by Jump. It allows annotations to be applied in a controlled UML standard-compliant way as the generated stereotypes extend exactly the required UML meta-classes. From a language engineering perspective, stereotypes facilitate defining constraints and model operations because they can directly be used as explicit types similar to a meta-class in UML. Jump realizes a mapping between Java’s annotation language and UML’s profile language. It enables the generation of specific stereotypes for corresponding annotations, which in turn leverage platform-specific profiles. As a basis for our generative approach, we employ model transformation techniques [19]. As a result, it allows engineers to “jump” from Java libraries to UML profiles. We collect all the generated profiles and make them publicly available in terms of the *UML-Profile-Store* [74], thereby complementing OMG’s collection of standardized profiles with supplementary profiles for the Java platform.

This article is an extension of our paper [6] at the MoDELS 2014 conference. We introduce three main extensions over the previous conference version. First, we consider novelties of Java 8 regarding *repeating annotations* as it leads us to revisit how stereotypes are defined and applied in profile applications [50], while leaving *type annotations* as subject for future work. We discuss pros and cons of three significantly different solutions to support *repeating stereotypes* in analogy to *repeating annotations* and modifications required to the current UML 2.4 formal specification and its Eclipse-based reference implementation that are implied by two of them. Second, we improve our previously introduced mapping to support the generation of profiles with repeating stereotypes. Moreover, we briefly discuss the contribution of the *UML-Profile-Store* to the Eclipse UML Profiles Repository (UPR) [75]. Our aim is to share all generated UML profiles with the Eclipse modeling community. Third, to strengthen the evaluation of our approach, we report on the scalability of Jump by providing performance measures of applying it to large Java code bases and demonstrate its practicability by applying it to a modernization scenario including both RE and FE processes.

**Fig. 1:**
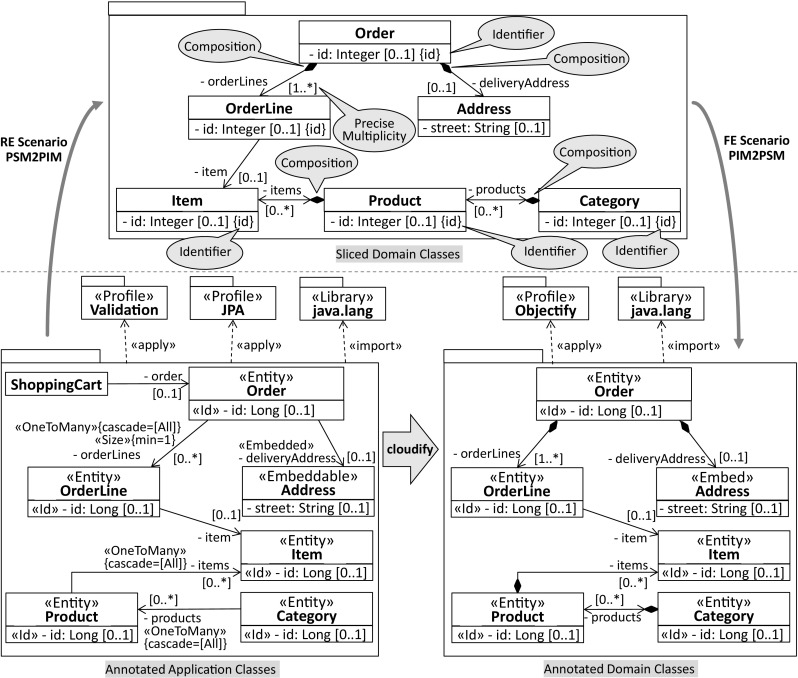
Typical Jump use case

The remainder of this paper is structured as follows. In Sect. 2, we motivate the practical value of platform-specific profiles by a typical Jump use case and give the background for *UML Profiles* and *Java Annotations* in terms of metamodels. Then, in Sect. 3, we discuss how repeating stereotypes may be introduced into UML. We present Jump in Sect. 4 by providing insights into our proposed conceptual mapping and elaborating effective solutions to overcome existing heterogeneities between Java and UML. In Sect. 5, we discuss our prototypical implementation based on Eclipse and its contribution to the Eclipse UPR, while in Sect. 6, we evaluate Jump. In particular, we (1) compare our methodology how to represent annotations and annotation types in UML with methodologies used in current UML tools, (2) evaluate the quality of automatically generated profiles compared to profiles used in practice, (3) show that Jump scales for Java libraries used in practice, and (4) report on our experiences applying Jump to a modernization scenario. Finally, in Sect. 7, we discuss related work and conclude in Sect. 8 with an outlook on future work.

## Motivation and background

Java annotations and UML profiles can be considered as general injection mechanisms for varying purposes. For instance, UML profiles are used to specify variation points of general UML semantics, introduce classifiers in addition to the standard UML classifiers, explicitly document design decisions, and capture platform-specific terminology. To motivate the practical value of platform-specific profiles that are generated from annotation-based Java libraries, we introduce a typical Jump use case. Then, we discuss the concepts of Java’s annotation mechanism and briefly introduce UML’s profile mechanism to establish the basis for our approach.

### Application of platform-specific UML profiles

A typical Jump use case is linked to scenarios in the setting of reverse engineering and forward engineering. These use cases are of particular relevance to migration projects, which aim at reinterpreting existing reengineering processes [44] in light of advanced model-based software engineering approaches [30]. In this respect, UML profiles play an important role as they enable the annotation of models with platform-specific information [66]. To demonstrate a concrete use case, we selected the JPA and Objectify profile from the area of data modeling. The idea is to replace the former profile by the latter one, thereby realizing a change of the data access platform as typically required by “moving-to-the-cloud” scenarios. Figure 1 depicts an excerpt of the PSMs of a typical eCommerce web application, where the platform refers to the selected profiles. From the JPA-based PSM, a sliced PIM is generated that sets the focus solely on the domain classes, i.e., annotated with JPA stereotypes, which are intended to be modified. Even better, this generated PIM interprets JPA stereotypes in terms of native UML concepts. As a result, the accuracy of the PIM is improved because it explicitly captures *identifiers*, *compositions*, and more precise *multiplicities*. These improvements in the PIM demonstrate the practical value of considering platform-specific information in the context of a model-based RE scenario. Furthermore, they leverage the refinement of the PIM toward an Objectify-based PSM without the need to identify mappings between the pertinent platforms. From the produced Objectify-based PSM, program code can be generated by also interpreting applied stereotypes in the context of a FE scenario. For instance, method bodies for CRUD operations can be generated for domain classes as they are indicated by the respective stereotypes and generated code elements can be automatically annotated. Clearly, Jump acts as an enabler for both RE and FE scenarios by providing the required platform-specific profiles.

### Representation of Java annotations in UML

Currently, three significantly different solutions exist to support Java annotations for UML models: The *built-in* annotation feature of modeling tools is used, a *generic* profile for Java is provided, which enables capturing annotations and their type declarations, and profiles are offered, which are *specific* to a Java library or even an application with custom annotation type declarations. The first solution is certainly the most generic one as it goes beyond Java and UML. Clearly, it facilitates capturing Java annotations, though the type declaration of an annotation in terms of a UML element and its application are not connected. A generic profile for Java emulates the representational capabilities of Java’s annotation language. Although with this approach the connection of annotation type declarations and their applications can be ensured, the native support of UML for annotating elements with stereotypes is still neglected. However, stereotypes specifically defined for annotation types would facilitate their application in a controlled UML standard-compliant way as they extend only the required UML meta-classes. From a language engineering perspective, such stereotypes facilitate defining constraints and model operations, such as model analysis or transformations, because they can directly be used in terms of explicit types similar to a meta-class in UML. Jump is based on a conceptual mapping between Java’s annotation language and UML’s profile language [6], which enables the generation of specific stereotypes for corresponding annotation types that in turn leverage platform-specific profiles.

**Fig. 2:**
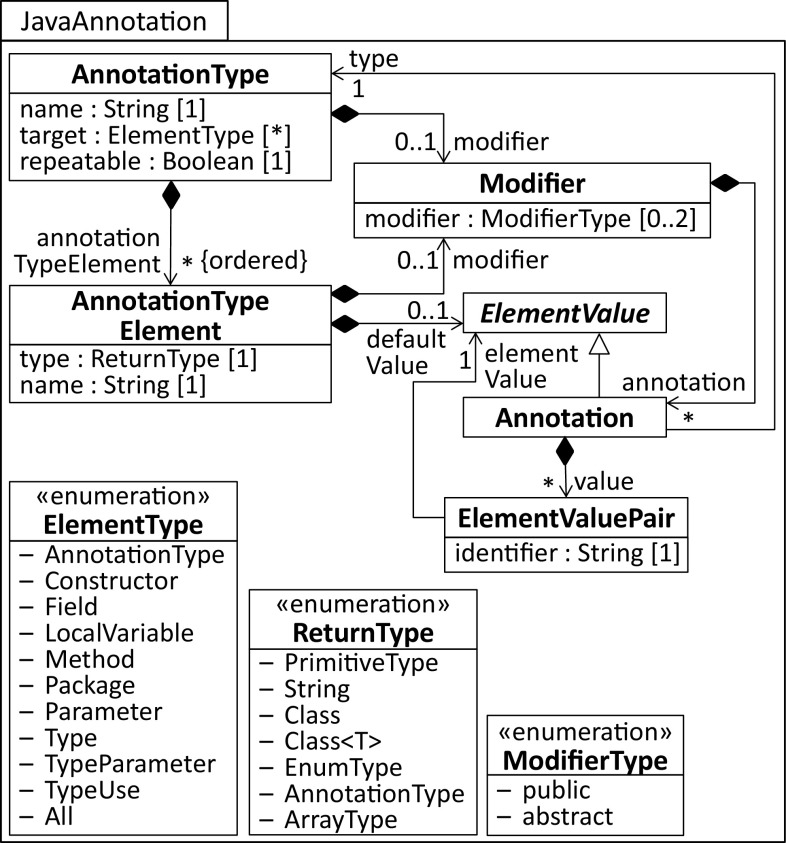
Metamodel of Java annotations

### Java annotations and UML profiles

Before annotations can be applied on code elements, they need to be declared in terms of annotation types. A rough overview of the main concepts behind annotations in Java is given in the metamodel depicted in Fig. 2. We extracted this metamodel from the JLS8 [64]. AnnotationTypes declare the possible annotations for code elements and may have, similar to Java interface declarations, optional modifiers. They are identified by a name. AnnotationTypes may themselves be subject for annotations. An Annotation references to its type and composes ElementValuePairs. They capture values passed to an annotation. Most importantly for the context of this work is the target annotation that is represented in the metamodel as an attribute for simplicity reasons. It is a meta-annotation because it can only be applied to declared annotation types to indicate the code elements that are valid bases for an application of an AnnotationType. The set of valid bases are captured by the literals of ElementType enumeration. Note that we omitted the newly introduced TypeUse and TypeParameter literals as they are considered as part of future work. Generally, UML does not support such annotations by default as it would require to extend not only meta-classes but also meta-features, which is not yet supported. The body of an annotation type declaration consists of zero or more AnnotationTypeElements for holding information of AnnotationType applications. They are declared in terms of method signatures with optional modifiers, a mandatory return type and name, and an optional default value that is returned if no custom value is set. The default value needs to conform to the defined return type of the AnnotationTypeElement. For instance, if the defined return type is AnnotationType, the default value needs to be an Annotation, which inherits from ElementValue. This abstract meta-class is specialized by other meta-classes, e.g., ConditionalExpression to support the non-array ReturnTypes and ElementValueArrayInitializer to support one-dimensional arrays thereof. For the sake of brevity, these additional specializations of ElementValue are omitted.

**Fig. 3:**
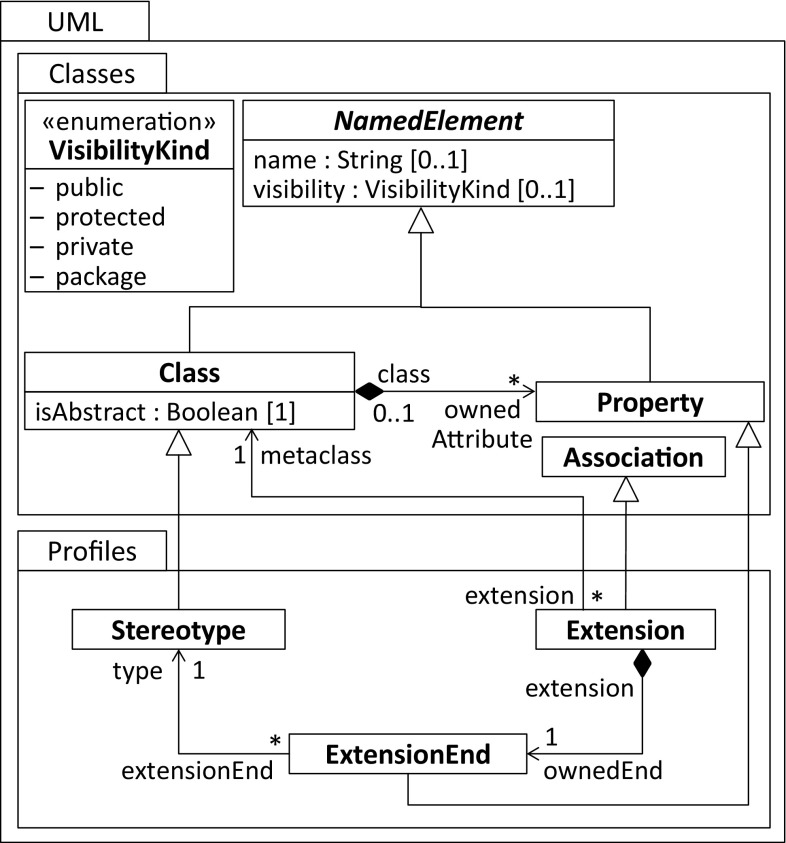
Metamodel of UML profiles

With the introduction of UML 2, the profile mechanism has been significantly improved compared to the beginnings of UML [31]. In particular, a profile modeling language has been incorporated in the UML language family to precisely define how profiles are applied on UML models and how stereotypes are applied to elements of those models. Figure 3 depicts the core elements of UML’s Profiles package and relates them to the Classes package of UML^2^.

A Stereotype is a specific meta-class used to extend meta-classes of the UML metamodel. This enables platform-specific concepts to be injected into instances of meta-classes that are extended by a defined stereotype. The Stereotype meta-class specializes the meta-class Class. Hence, it inherits modeling capabilities such as properties. Similar to AnnotationTypes, an instance of a Stereotype is identified by a name and modified by an optional visibility and the mandatory isAbstract property. A defined stereotype references the extended meta-classes via instances of the Extension relationship. The Extension relationship inherits from the Association meta-class. As a result, it is a binary relationship with two association ends where both are realized by a Property. The property that points to the extended meta-class is contained by the defined stereotype, whereas the extension contains the other association end. It realizes the reference from the extended meta-class back to the defined stereotype. This back reference is represented by the ExtensionEnd meta-class, which inherits from Property.

### Defining stereotypes for declared annotations types

To demonstrate the relationship between annotations and stereotypes, we set the focus on the Order class of the JPA-based PSM in Fig. 1. Listing 1 shows the *application* of the Entity annotation type to the Order class, whereas Listing 2 depicts the respective *declaration* at the programming level.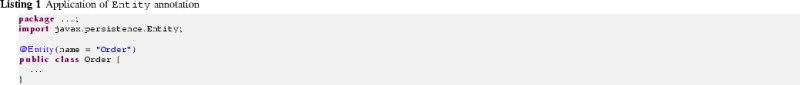 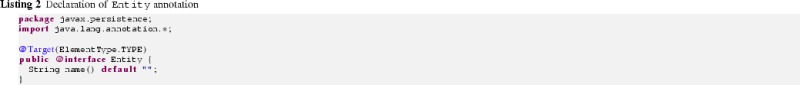


**Fig. 4:**
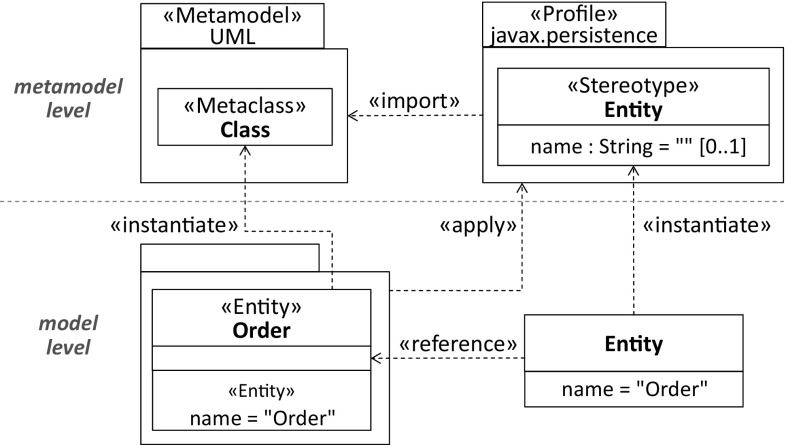
Application of Entity stereotype

**Fig. 5:**
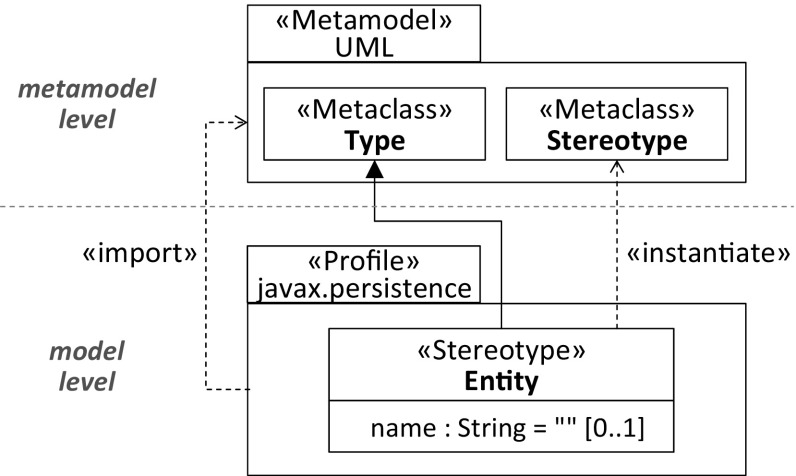
Declaration of Entity stereotype

The corresponding UML representations are shown in Figs. 4 and 5. They demonstrate the stereotype application to the Order class and the Entity definition by a stereotype.

Considering the former, the UML profile which covers the Entity stereotype needs to be applied to the Order’s package as a prerequisite for the stereotype application. Applying a stereotype means that it is instantiated similar to any other meta-class that is used to create elements on the model level, e.g., the Order class which is an instance of the meta-class Class. Hence, a declared stereotype can be considered as part of the metamodel level if the focus is on the stereotype application [4]. A stereotype instance references the element on the model level to which the respective stereotype has been applied. In our example, the Order class is thus referenced by an instance of the declared Entity stereotype.

Considering the declaration of the Entity stereotype, it comprises as expected the name property corresponding to the annotation type element of the Entity annotation type. To ensure that it provides at least similar capabilities as the *Entity* annotation type, the Extension relationship references the UML meta-class Type.

Since Java 8, *repeating annotations* enable the same annotation to be repeated multiple times in the place where it is declared. Obviously, this repeatable application of annotations has an effect on determining the multiplicity of the ExtensionEnd contained by the Extension relationship. In case of repeating annotations, the multiplicity should be 0..*, which expresses that the corresponding stereotype can also be applied to base elements^3^ multiple times. However, the UML standard introduces an OCL constraint, see page 683 of the standard [61], that explicitly hinders the application of the same stereotype to the same base element more than once. As shown in Listing 3, the OCL constraint restricts the upper bound of the extension end to 1.
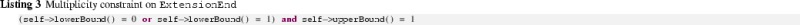


When considering a stereotype as a means to *classify* base elements, the restriction on the upper bound of the extension end seems reasonable. Classifying the same base element twice by the same stereotype is obviously inappropriate. In contrast, when considering a stereotype as a means to *annotate* base elements, there are use cases for applying the same stereotype to a base element multiple times. For instance, in the context of model versioning dedicated stereotypes can be used to visualize changes to a model element, e.g., “update class,” and highlight potential conflicts, e.g., contradicting updates to a class, as a result of concurrently edited model versions [12]. As updates to classes may be manifold, the respective stereotype is ideally applied to the changed class several times where each atomic change is captured by exactly one applied stereotype. To give another example, expressing several queries for an entity with the JPA profile require to apply the NamedQuery stereotype multiple times. As a result, even though *repeating stereotypes* in analogy to repeating annotations are currently not supported by standard UML, they are still desirable.

## Repeating stereotypes

To realize repeating stereotypes, several solutions are conceivable. Table 1 summarizes three such possible solutions and shows pros and cons for all of them. Concerning the UML metamodel and tools that depend on it, we refer to the Eclipse-based reference implementation.

**Table 1:** Possible solutions for repeating stereotypes

Solution	Stereotype		Changes in UML metamodel and tools	Backward compatibility	
	Repeatable application	Container		UML metamodel	Tools
Composition of multiple stereotypes	Not supported only contained by a dedicated stereotype	Explicitly modeled	Not required	Yes	Yes
Emulation of repeating stereotypes	Supported but contained by a dedicated stereotype	Automatically generated	Yes, moderate effort	No	Yes
Native support for repeating stereotypes	Supported	Not required	Yes, relatively high effort	No	No

The first solution is fully compliant to the current UML standard. In fact, it does not actually apply several stereotypes to a base element. Instead, a dedicated stereotype acts as a container for the repeating stereotypes. This solution foresees that the container stereotype is explicitly created by the modeler. As a result, changes to the UML metamodel and tools built on top of its API are not required because the repeating stereotypes are only referenced by their container stereotypes rather than applied to base elements. On the contrary, however, standard operations, for instance, to apply stereotypes and retrieve them, are not applicable by this solution for repeating stereotypes as they provide the expected result only for stereotypes that are applied following the standard procedures. This drawback is compensated by the second solution. It emulates repeating stereotypes as a result of slight modifications to the operations provided for stereotypes. Even though, similarly to the first solution, a container stereotype is exploited also by the second solution, this container is automatically generated on demand. Moreover, as a result of the modifications required by this solution, all standard operations for stereotypes are applicable also to repeating stereotypes. However, the extension ends pointing to them need to be multivalued to ensure that they can be applied multiple times. Consequently, this solution neglects the multiplicity constraint of the ExtensionEnd meta-class, which in turn leads to profiles that do not fully conform to the current UML metamodel. Still, the compatibility with existing tools can be ensured because the required changes can completely be hidden by the UML metamodel API. This backward compatibility cannot be maintained by the third solution. To natively support repeating stereotypes without providing a dedicated container stereotype requires not only changes in the UML metamodel API but also how they are represented and edited by the tools. For instance, applied stereotypes are represented according to unique categories to which also their features are assigned, where the category is derived from the name of a stereotype. Applying the same stereotype multiple times to the same base element would result in a single category to which all the features of the applied stereotypes are assigned.

To demonstrate how the profiles with repeating stereotypes of the three discussed solutions differ from each other, we refer again to the NamedQuery annotation of the JPA. Listing 4 shows its declaration as a repeatable annotation, whereas Listing 5 declares the required container annotation. For compatibility reasons, in Java 8, repeating annotations are stored in a container annotation that is automatically generated by the Java compiler once the annotation is applied.
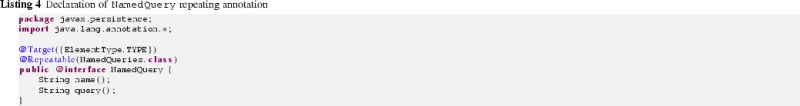
 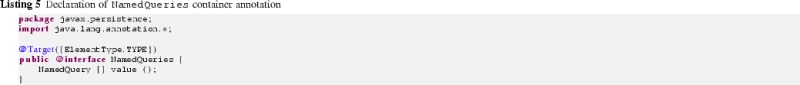

**Fig. 6:**
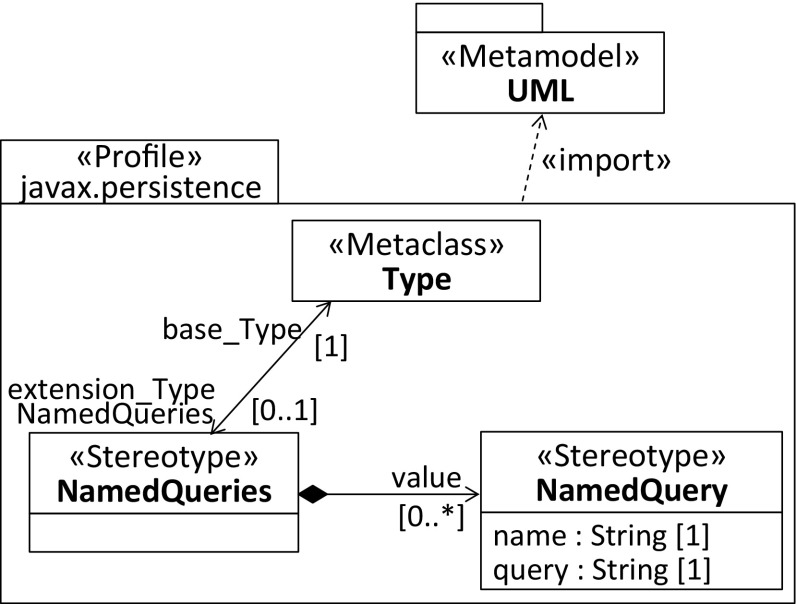
Profile for composing multiple stereotypes, see first solution in Table 1

**Fig. 7:**
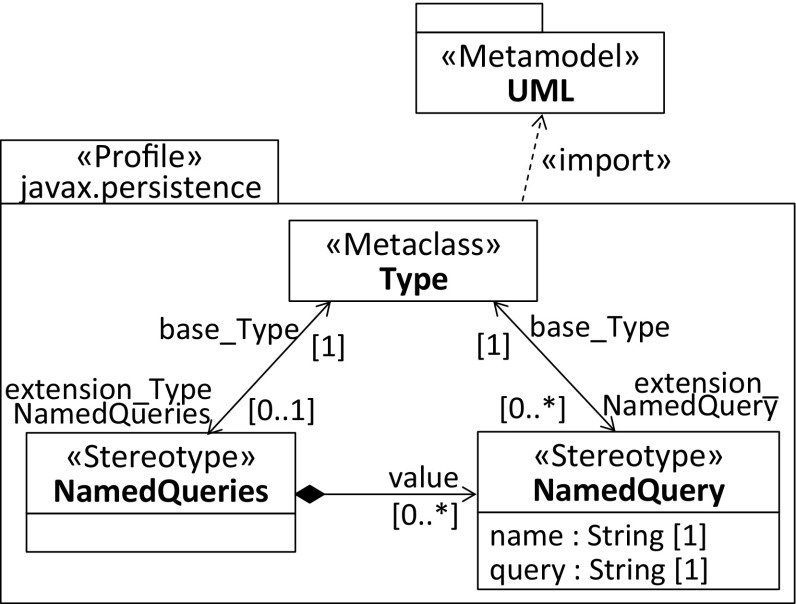
Profile for emulating repeating stereotypes, see second solution in Table 1

**Fig. 8:**
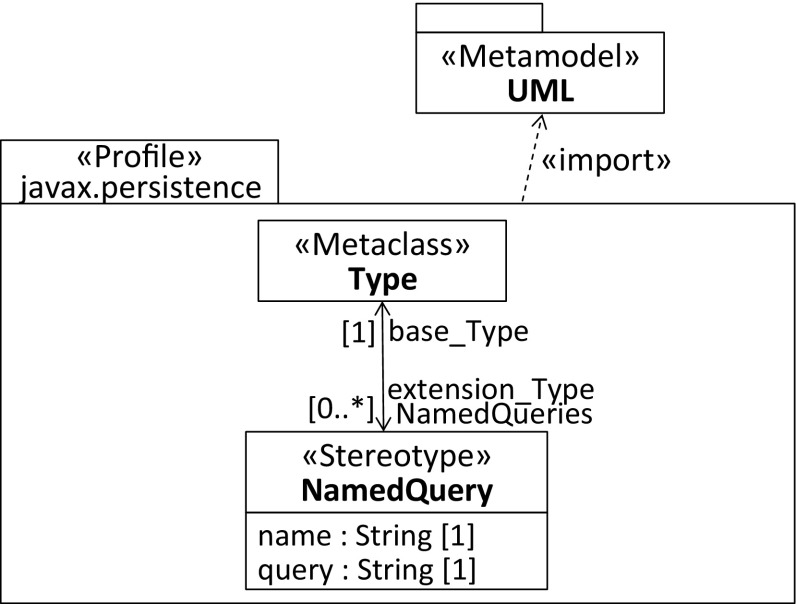
Profile for native support of repeating stereotypes, see third solution in Table 1

Considering the possible profile solutions in Figs. 6,  7, and 8 for the annotation declarations, we selected the notation used to represent associations and their member ends instead of extensions [50] to explicitly indicate the multiplicities of the extension relationships. The first profile depicted in Fig. 6 allows multiple NamedQuery stereotypes to be composed by its container stereotype. As expected, the latter extends Type, where the multiplicity of the extension end pointing to the NamedQueries stereotype is 0..1. It indicates that the container stereotype can be applied once, which is sufficient because the composition relationship between the NamedQueries stereotype and the NamedQuery stereotype is multivalued.

Similarly, the second profile shown in Fig. 7 exploits a multivalued composition relationship to emulate repeating stereotypes. The main difference compared to the first profile is that the NamedQuery stereotype also extends Type, where the multiplicity of the extension end pointing to the stereotype is 0..*, which indicates that it is a repeating stereotype. As a result, the NamedQuery stereotype is applicable to base elements that are instances of the meta-class Type. It is important that the extension relationships of both defined stereotypes point to the same meta-class because the container stereotype needs to be applicable to exactly the same set of base elements as the repeating stereotype. In fact, this solution resembles the realization of repeating annotations in Java 8. From the perspective of a modeler, the second profile is more powerful compared to the first one, as the required container stereotype is managed in the background by the UML metamodel API and hence fully transparent to the modeler. The development effort is slightly higher and the profile more complex because an additional extension relationship is required for the repeating stereotype.

Finally, the profile envisaged for the native support of repeating stereotypes is shown in Fig. 8. It does not require a container stereotype to capture repeating stereotypes because they are assumed to be directly applied to the base elements. The main difference to the previous solution is that each applied repeating stereotype is captured by its own StereotypeApplication instead of composed by an artificially introduced container stereotype for reasons of backward compatibility. The latter can be considered as the trade-off between natively supporting repeating stereotypes and guaranteeing that the solution is compatible at least with tools that are built on top of the UML metamodel API.

Concerning support for the three discussed solutions of repeating stereotypes and the pertinent profiles, Jump allows the generation of these profiles by passing the respective configuration option. Hence, the modeler can decide which profile version should be generated from a Java library. Clearly, to emulate repeating stereotypes a modified UML metamodel API is required, whereas native support for them requires also modifications in the tools built on top of this API. We consider support for a native realization of repeating stereotypes as future work.

**Fig. 9:**
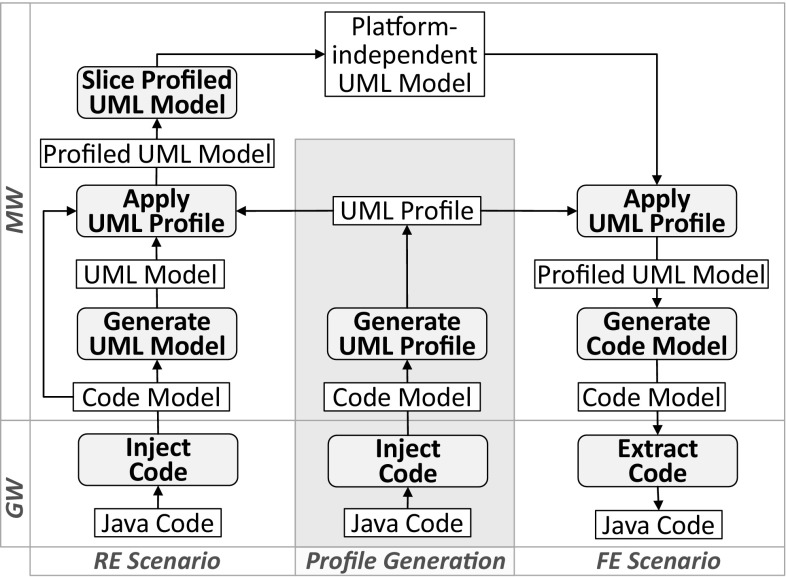
Processes for UML profile generation and application scenarios

## Generating UML profiles from Java libraries

We start our investigation for generating UML profiles from annotation-based Java libraries by presenting the process of Jump, as shown in Fig. 9. The entry point to the profile generation is *Java Code* that is translated into a what we call *Code Model* conforming to MOF [58]/EMF [21]. As a result of this first step, the transition from a text-based representation into a model-based representation is accomplished. The generated *Code Model* is a one-to-one representation of the *Java Code* and the basis for generating a *UML Profile*, which captures Java annotation type declarations in terms of UML stereotypes (see middle of Fig. 9). They serve as foundation to exploit profiles as an annotation mechanism [71].

In case of the RE scenario, a *Code Model* is generated in a first step similar to the UML profile generation. The *Profiled UML Model* is generated from the *Code Model* by taking into account profiles that provide stereotypes corresponding to annotations in the *Code Model* (see left hand side of Fig. 9). Annotated elements of the *Code Model* indicate the elements of the *Profiled UML Model* to which those stereotypes need to be applied. As shown in Fig. 1, stereotypes applied to elements of the *Profiled UML Model* may lead to a more accurate *Platform-independent UML Model* if they are appropriately interpreted by a model slicer [9]. This slicing step is specific to the interpreted stereotypes and hence the profiles that cover them, whereas the steps of generating a *Profiled UML Model* and an intermediate *Code Model* are completely generic in the sense that any Java application and library can be translated into a *Profiled UML Model*.

A *Profiled UML Model* is accomplished in the case of the FE scenario (see right hand side of Fig. 9) by applying profiles to a *Platform-independent UML model*. While UML’s profile mechanism is generic in the sense that arbitrary profiles can be applied to a UML model, automating the application of stereotypes to particular elements is certainly specific to the application scenario. In contrast, both the generation of the *Code Model* and the extraction of the *Java Code* are generic provided that the employed code generation facility supports stereotypes.

Considering the generation of stereotypes compared to the RE and FE scenarios where those stereotypes are applied, the respective profile generation process operates at a different level as the processes supporting the two application scenario because declared stereotypes can be considered as part of the metamodel level instead of the model level (see Sect. 2.4). Following this classification into different levels, the profile generation is a meta-level process, which produces elements at the metamodel level.

Finally, bridging the two technical spaces [41] we are confronted with, i.e., GrammarWare (GW) [45] and ModelWare (MW) [48], is required for the two scenarios as well as Jump.

### Bridging technical spaces

Transforming plain Java code into a UML-based representation requires overcoming the different encoding and resolving language heterogeneities. Concerning the first aspect, the Java code needs to be encoded according to the format imposed by the modeling environment [7]. Concerning the second aspect, a bridge between Java and UML based on translations requires a conceptual mapping between the two languages. Instead of directly translating plain Java code into a UML-based representation, the use of a two-step approach is preferable [37], which is also applied by Jump. In a first step, *Java Code* is translated into a *Code Model* that uses Java terminology and structures conforming to the Java metamodel provided by MoDisco [14]. This *Code Model* is the basis for generating UML profiles and input for the second step that is dedicated to resolving language heterogeneities by relying on the correspondences between the Java and UML metamodels.

### Generating UML profiles

To facilitate the generation of UML profiles, we present a conceptual mapping between Java’s annotation language and the profile language of UML. Therefore, stereotypes play a vital role for representing annotation types on the model level as they enable their application in a controlled UML standard-compliant way. From a language engineering perspective, stereotypes only extend the required UML meta-classes and facilitate defining constraints and model operations, such as model analysis or transformations, because they can directly be used in terms of explicit types similar to a meta-class in UML. Our proposed mapping is generic in the sense that any declared annotation type can be represented by a stereotype.

**Table 2:** AnnotationType to Stereotype mapping

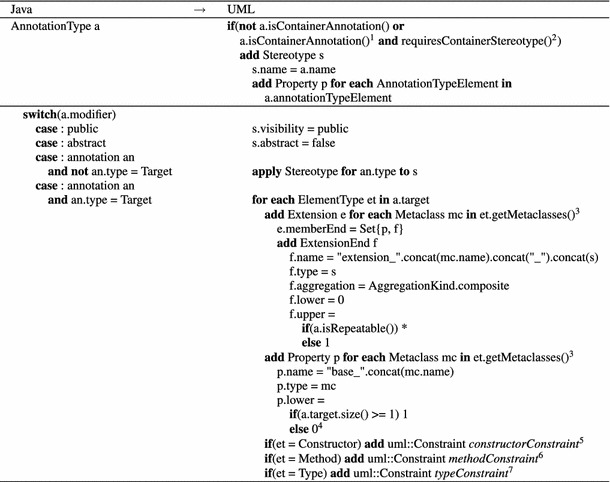	

$^{1}$ See Listing 6 for further details

$^{2}$ It provides a boolean value of the decision taken by the modeler regarding repeating stereotypes

$^{3}$ See Listing 7 for further details

$^{4}$ AnnotationTypes that are intended to used only inside other annotations require a zero multiplicity (e.g., QueryHint of the JPA)

$^{5}$ See Listing 8 for its specification

$^{6}$ See Listing 9 for its specification

$^{7}$ See Listing 10 for its specification

#### AnnotationType $\rightarrow$ Stereotype

The mapping presented in Table 2 provides the basis to generate an applicable Stereotype from an AnnotationType. First of all, it needs to be decided if a stereotype should be generated at all, because container annotations required to declare repeating annotations may not in any case result in a corresponding container stereotype as discussed in the previous Sect. 3. For that reason, a corresponding container stereotype is generated from a container annotation only if it is required, which depends on the provided configuration parameter passed by the modeler. Whether an annotation type is exploited as a container annotation is indicated by the meta-annotation Repeatable applied to the annotation type for which the container annotation is declared. Listing 6 gives the respective function to check for container annotations.
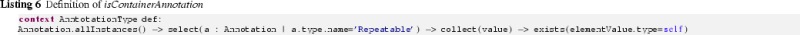


In cases where repeating stereotypes should be composed by a dedicated stereotype instead of directly applied to a base element, see first row in Table 1, or emulated in terms of repeating annotations, see second row in Table 1; a container stereotype is generated. Otherwise, its generation is neglected; see third row in Table 1. The function *requiresContainerStereotype* provides a boolean value of the decision taken by the modeler.

Generally, the generation of a stereotype from an annotation type requires not only its signature to be considered, but also Java’s Target meta-annotation. It determines the set of code elements an annotation type is applicable to. The name and, with two exceptions, the defined modifiers of an AnnotationType can straightforwardly be mapped to UML. First, the abstract modifier would lead to Stereotypes that cannot be instantiated if directly mapped. The problem is caused by Java’s language definition. Although the abstract modifier is supported to facilitate one common type declaration production rule, it does not restrict the application of AnnotationTypes. To ensure the same behavior on the UML level, we never declare a Stereotype to be abstract. Second, because annotations are considered as modifiers, it needs to be ensured that the Target annotation is properly treated. In fact, the defined set of Java ElementTypes determines the required set of Extensions to UML meta-classes that specify the application context of the stereotypes. Listing 7 defines the correspondences between Java ElementTypes and UML meta-classes.
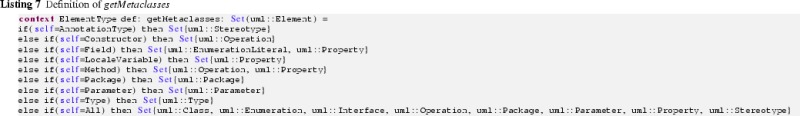


Most Java ElementTypes correspond well to UML meta-classes. Still, some constraints are required to precisely restrict the application scope of the generated Stereotype according to their intention. UML does not explicitly support a constructor meta-class. The workaround is to map the Constructor to Operation and introduce a constraint that emulates the naming convention for constructors in Java, as depicted in Listing 8. Note that annotation types can have several target types. Thus, before validating the OCL constraint, we have to check which target is actually used in the application.
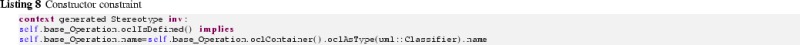

**Table 3:** AnnotationTypeElement to Property mapping

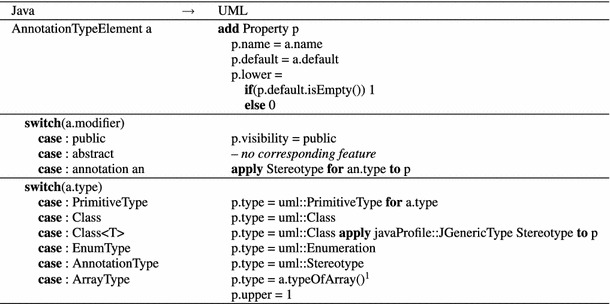	

$^{1}$ Extracts the type of the array

Similarly, the mapping of Java methods to UML requires a constraint as a declared method of an AnnotationType, i.e., AnnotationTypeElement, is mapped to a Property rather than an Operation in UML. This is because such methods do not provide a custom realization but merely return their assigned value when they get called. Properties in UML provide exactly this behavior. Hence, the constraint in Listing 9 ensures that stereotypes generated from annotation types that target Java methods are applicable also to Property if they are contained by a Stereotype.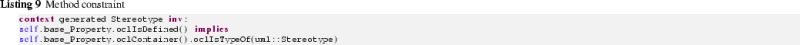 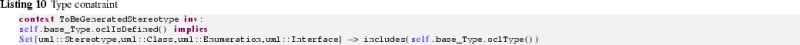


Finally, we use a constraint to overcome the heterogeneity of Java’s and UML’s scope of Type. Consequently, stereotypes that extend Type are constrained to those elements that correspond to the set of elements generalized by Java’s Type: AnnotationType, Class, Enumeration, and Interface. The clear benefit of this approach is a smaller number of generated extension relationships between stereotypes and meta-classes in the profile. The constraint is depicted in Listing 10.

#### AnnotationTypeElement $\rightarrow$ Property

An AnnotationTypeElement is mapped to aProperty as depicted in Table 3. Except for the fact that UML properties cannot be defined as abstract,AnnotationTypeElements straightforwardly correspond to Properties. In Java, AnnotationTypes cannot explicitly inherit from super annotations. Therefore, the abstract modifier is rarely used in practice. To fully support all return types of AnnotationTypeElements, we introduce a Stereotype to address the generic capabilities of java.lang.Class, which is not the case for UML’s meta-class Class. Hence, we apply our custom JGenericType stereotype to properties with return type ${\texttt {Class<T>}}$.

## Implementation and collected UML profiles

To show the feasibility of Jump, we implemented a prototype based on the Eclipse ecosystem. We developed three transformation chains— *JavaCode2UMLProfile*, *JavaCode2ProfiledUML*, and *ProfiledUML2JavaCode*—to realize Jump and the RE and FE processes are shown in Fig. 9. For injecting Java code, we employed MoDisco [14]. Hence, Jump can be considered as a model discoverer to extract UML profiles from Java libraries. To realize the FE scenario, we extended the Java-based transformer provided by Obeo Network^4^. The prototype and the collection of profiles that we have generated for the evaluation of Jump are available at the *UML-Profile-Store* [74]. It covers 20 profiles, comprising in total over 700 stereotypes. To share these profiles with existing community portals, we submitted them also to ReMoDD [29]. Since early 2014, an Eclipse project is dedicated to develop a centralized repository that hosts standardized UML profiles, such as SoaML [59]. The *UML-Profile-Store* complements the set of standardized profiles of the UML Profiles Repository (UPR) with profiles specific to the Java platform. Contributing these Java-specific profiles to the UPR appears obvious. Therefore, the Eclipse modeling community can access them via a common repository for standardized platform-independent profiles as well as profiles that are specific to a platform such as Java.

## Evaluation

The evaluation of Jump is fourfold. First, we compare it with existing modeling tools regarding their representational capabilities for dealing with the declaration and application of Java annotation types. Second, we compare UML profiles automatically generated by Jump with UML profiles delivered by IBM’s Rational Software Architect. Therefore, our focus is on estimating the quality of the generated UML profiles. Third, to show that Jump scales we report on its application to different Java libraries which are widely used in practice. Finally, we demonstrate the practical relevance of Jump in the context of a modernization scenario to the cloud.

- **RQ1**: *What are the methods of current modeling tools to represent Java annotation types and their applications in UML and what are the practical implications?*
- **RQ2**: *How is the quality of UML profiles automatically generated from annotation-based Java libraries compared to UML profiles used in practice?*
- **RQ3**: *Does Jump scale for Java libraries and applications used in practice?*
- **RQ4**: *How can developers benefit from JUMP by applying it in a modernization scenario?*

In the following, we are going to answer these four research questions *RQ*1–*RQ*4 in the Sects. 6.1–6.4.

### Methodological evaluation

As several commercial and open-source modeling tools provide modeling capabilities for UML and the Java platform, the aim of this study is to investigate on their methods for dealing with the application and declaration of annotations. For that reason, we set the focus on a Java-based reverse engineering example that includes annotations and their declarations. We aim to answer (*RQ*1) by defining a set of comparison criteria that mainly address (1) how the conceptual mapping between Java and UML for annotations is achieved by current modeling tools and (2) the generative capabilities of these tools regarding profiles. Based on the defined criteria, we evaluate six representative modeling tools and JUMP.

#### Comparison criteria

As there are different approaches on how annotation types and their applications are represented on the model level, the first and the second comparison criteria (*CC*1 and *CC*2) refer exactly to these extensional capabilities. The third criterion (*CC*3) refers to the support of generative capabilities regarding profiles.

- *CC*1: How are Java annotations applied to UML models?
- *CC*2: How are Java annotation type declarations represented in UML?
- *CC*3: Is the generation of UML profiles from Java code supported?

#### Selected tools

We selected six major industrial modeling tools that claim to support reverse engineering capabilities for Java and UML, as summarized in Table 4.

**Table 4:** Comparison results

Modeling Tool			Mapping (Java -$>$ UML)		UML profile generation
Name	Version	Availability	Annotation application	Annotationdeclaration	
Altova UML	2015	Commerical and free for academic use	Generic Java profile	Interface	$-$
ArgoUML	0.34	Open-source	Generic Java profile	Interface	–
Enterprise architect	9.3	Commerical and free for academic use	Built-in tool feature	Interface	$-$
Magic draw	18.0	Commerical and free trial version	Generic Java profile	Interface	$-$
Rational software architect	8.5.1	Commerical and free for academic use	Specific profiles	Stereotype	$-$
Visual paradigm	12.1	Commercial and free community edition	Built-in tool feature	Class	$-$
JUMP	1.1.0	Open-source	Specific profiles	Stereotype	+

#### Evaluation procedure

We defined a simple reference application [74] that declares a Java class to which we applied an annotation type from an external library. For the purpose of importing the application, we activated the offered functionality of the modeling tools required for a reverse engineering scenario from Java to UML. While some of the modeling tools are delivered with standard configurations, other modeling tools allow configurations to change the reverse engineering capabilities by using specific wizards. Moreover, some modeling tools go one step further and allow modifications on the transformation scripts used for the import of Java code. We evaluated the capabilities of the modeling tools offered in the standard settings and explored the different wizard configurations if supported, but we restrained from modifying transformation scripts.

#### Results

The results of our comparison are summarized in Table 4. It shows that the investigated tools apply one of the three significantly different approaches to represent Java annotations in UML. We have already discussed these approaches and their pros and cons in Sect. 2.2. While all evaluated modeling tools support the generation of annotated UML class diagrams from Java applications, none of them is capable of generating profiles dedicated to Java libraries. Only the Rational Software Architect also exploits the powerful capabilities of stereotypes and profiles for capturing declared Java annotation types.

### Quality evaluation

As UML profiles are already offered by current modeling tools, the aim of this study is to investigate their quality in comparison with profiles automatically generated by Jump. For that reason, we conducted a positivist case study [51] based on real-world Java libraries to evaluate the commonalities and differences between generated profiles and profiles used in practice by following the guidelines of Roneson and Hörst [70]. We aim to answer (*RQ*2) by defining the requirements of the case study, briefly mention the used Java libraries, and specify the measures based on which the comparison is conducted. Then, we discuss the results of our study not only from a syntactic perspective, but also from a semantic one. The rationale behind this two-step approach is that even though a syntactical matching process for comparing the profiles provides already valuable results, some interesting correspondences may still be uncovered because of potential syntactical and structural heterogeneities [79] between the compared profiles and the conservative matching strategy applied for the syntactical comparison.

#### Case study design

To conduct this study, the source code of Java libraries that exploit annotations is required. Furthermore, we require existing profiles that claim to support the selected Java libraries on the model level. To accomplish an appropriate coverage of different scenarios, the selected Java libraries ideally comprise different intrinsic properties with respect to the design complexity and exploited language elements. Unfortunately, profiles specific to Java libraries in reasonable quality are rarely available. Consequently, in the process of selecting the Java libraries for this study, we were also confronted with the actual offering of modeling tools. IBM’s Rational Software Architect (RSA) is obviously close to Jump and offers several profiles of well-known Java libraries mainly for code generation purposes. Thus, we conducted this study by relying on profiles of RSA in version 8.5.1. We selected four established Java libraries for which the source code is available and a corresponding RSA profile in the same major version is offered: Java Persistence API (JPA), Enterprise Java Beans (EJB), Struts and Hibernate. RSA offers them in a UML standard-compliant way. Consequently, we could directly compare them without an intermediate conversion step. All the case study data including the Java libraries and the profiles are available at our project web site [74].

#### Case study measures

The measures used in the case study are based on model comparison techniques [47]. Thus, we are interested in equivalent elements that reside in our generated profiles and in the RSA profiles, elements that reside in both solutions but still show differences in their features, and elements that are only available in one of the compared solutions. The measures for estimating the quality of the generated profiles are collected in a two-step matching process. While the first step automatically collects measures based on syntactic model comparison, the second step relies on manually processing differences produced in the first step to deal with semantic aspects.

In the syntactic model comparison, we compute the following measures for certain model elements. To determine element correspondences, we employ as matching heuristic name equivalence, i.e., only if two elements have completely the same name, they are considered to be corresponding. If an element has no name, such as the Extension relationship, it is considered that the elements are corresponding if their source and target elements correspond. Finally, fine-grained comparison of the feature values for the given elements is performed. Regarding model elements, we set the focus on (1) Stereotypes that are common to both and unique either to Jump or RSA, (2) differences regarding the Extensions of common Stereotypes, and (3) differences regarding the Properties such Stereotypes cover.

In the semantic model comparison, we take the syntactical differences as input and aim at finding additional correspondences between elements which are hardly explored by a pure syntactic comparison due to the conservative matching strategy. We investigate unmatched elements, especially stereotypes, in our generated profiles and in the RSA profiles, and reason about possible element correspondences beyond string equivalences. Finally, in the semantic processing, we further evaluate the correspondences found in the first phase due to the potential syntactical and structural heterogeneities.

#### Results

**Fig. 10:**
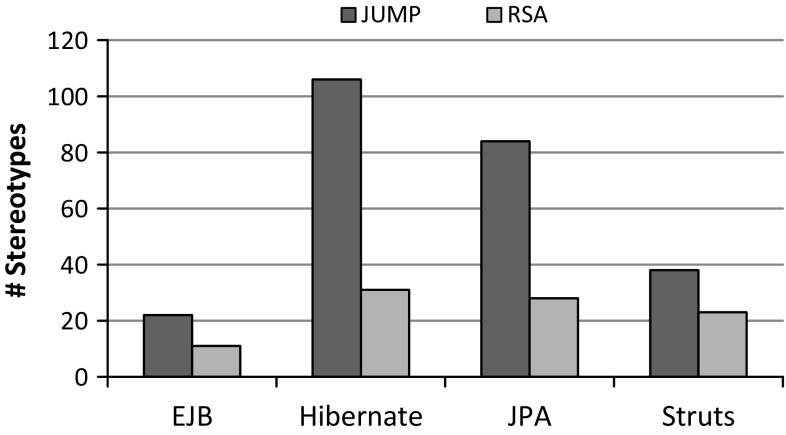
Absolute number of stereotypes

We now present the results of applying Jump to the selected Java libraries and compare them to the profiles offered by RSA. They are also available at our project web site [74]. The absolute number of generated stereotypes by Jump and the provided ones by RSA are depicted in Fig. 10.

**Fig. 11:**
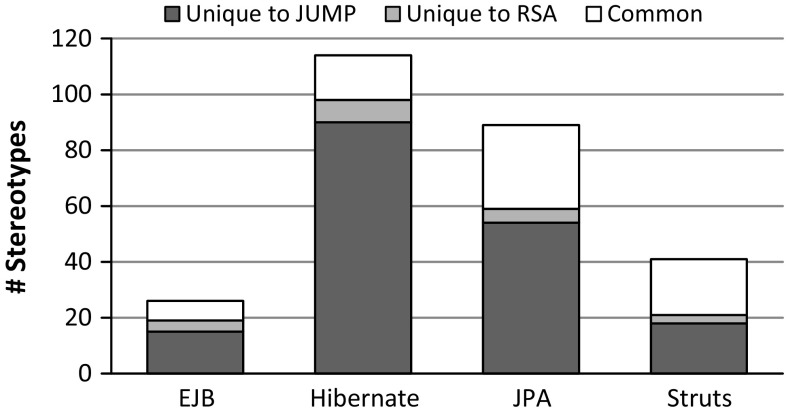
Comparison of stereotypes

**Fig. 12:**
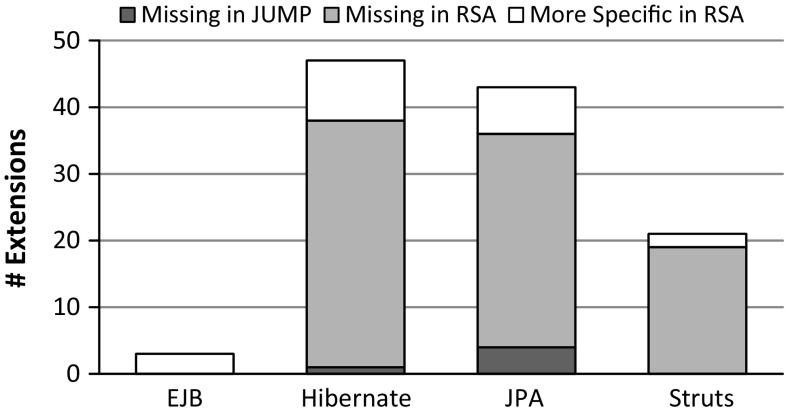
Comparison of extensions

Figure 11 shows (1) the number of stereotypes generated by Jump but not covered by the RSA profiles, (2) the number of stereotypes that are exclusively covered by the RSA profiles, and (3) the number of stereotypes that are common to both. These results include correspondences between stereotypes detected throughout the syntactic and semantic comparison. For instance, the EJB profile of RSA covers stereotypes that refer to the @Local and @Remote annotations of the EJB library, though their signature additionally contains the substring “Interface”. Another example refers to the class QueryHint in the JPA profile of RSA, which is in fact an annotation type in the JPA library. In our solution, the QueryHint is represented by a stereotype even though it is also valid to use a class instead, because the QueryHint cannot actually be applied, but can rather only be used inside of another annotation. Although some stereotypes in the set of common ones show differences regarding the meta-classes they extend, we granted them to be equal if the extended meta-classes are related by a generalization relationship. We encountered this case in the EJB and the JPA library with respect to extensions of the meta-classes Type and Class. Stereotypes generated by Jump extend the more general meta-class Type because the scope of Java’s element type Type also covers Enumeration, Interface, and AnnotationType in addition to Class.

The comparison regarding extensions of stereotypes common to both Jump and RSA is shown in Fig. 12. In a few cases, the RSA profiles comprise extensions to the UML meta-class Association to allow stereotypes on associations between elements rather than on properties contained by associations. Although both modeling variants are valid, we adhere to the second one as it is more accurate w.r.t. the target specifications of the original annotation type declarations.

**Fig. 13:**
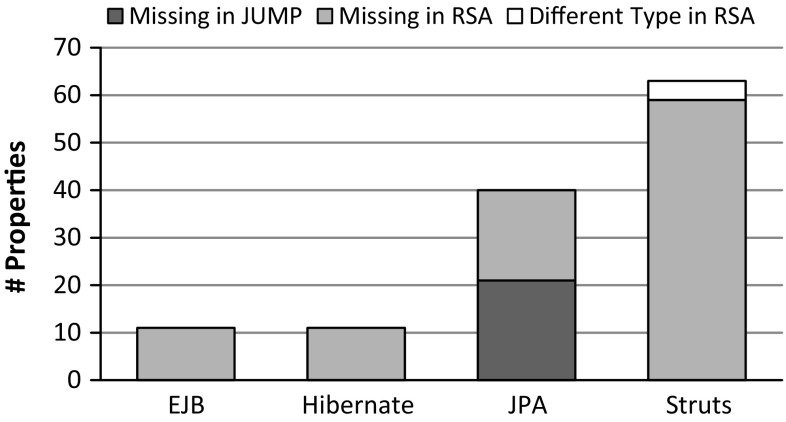
Comparison of properties

Finally, in Fig. 13, the differences regarding the properties of common stereotypes are presented. Except for the JPA profile, we cover all stereotype properties of the RSA profiles. Consequently, our profiles are more complete. The main reason for missing properties in our JPA profile seems to be that RSA provides additional properties for code generation purposes, but these properties are not covered by the JPA library.

#### Discussion

In this study, we have demonstrated that automatically generated UML profiles from Java libraries comprise a more comprehensive set of stereotypes and features compared to profiles used in practice for the purpose of supporting such libraries. Clearly, the purpose of the developed profiles plays an important role. From a forward engineering perspective, one may argue that the set of stereotypes which is actually supported by the accompanying code generators is reasonable to capture on the model level. In fact, RSA offers code generation capabilities specific to the profiles we have evaluated in this study. However, for unsupported annotations, which have no corresponding stereotypes, code generators may only produce program code by conventions without allowing developers to intervene in this generation process on the model level. From a reverse engineering perspective, we would lose relevant information on the model level if offered profiles provide less capabilities compared to the programming level, which is, however, the case for RSA profiles. Hence, with a fully automated approach, the quality of current profiles can be improved by providing more complete stereotypes that precisely capture the intention of the original annotation types in terms of target definitions, member declarations, and return values of such members.

#### Threats to validity

There are two main threats that may jeopardize the internal validity of this study. First, we consider only profiles from RSA. The main reason for this procedure is that RSA applies a similar approach as Jump and offers specific UML profiles for Java libraries. Furthermore, RSA offers standard-compliant UML profiles that conform to the same UML 2 metamodel implementation as used in Jump. Second, it may be possible that we missed correspondences between elements of the profiles involved in the study. Several kinds of heterogeneities [79] exist that are real challenges for model matching algorithms and, thus, may affect the results of our study. However, by applying a two-step matching process which includes a syntactic as well as semantic comparison phase, we tried to minimize the possibility of missing correspondences as a result of different naming conventions and modeling styles. While in the first phase we used a quite conservative matching strategy to avoid false positives, we applied a rather liberal strategy in the second phase to avoid losing potential correspondences.

**Table 5:** Performance measures: UML profile generation

Library	Size of input/output model	Appliedstereotypes	Executiontime in sec
EJB [25]	10K/1.5K	32	1.302
JPA [42]	20K/4K	84	2.165
Objectify [57]	40K/0,6K	20	1.442
Struts [73]	90K/2.5K	38	3.672
Hibernate [38]	300K/5K	108	12.042
Spring [72]	500K/3K	63	9.463
EclipseLink [22]	700K/6K	127	19.193

**Table 6:** Performance measures: profiled UML model generation

Application	Size of input/output model	Declared stereotypes	Execution time in sec
EJB [25]	10K/0.6K	5 (1 Profile)	1.647
Petstore (PS) [67]	10K/1.5K	287 (12 Profiles)	3.977
DEWS core [5]	30K/3K	253 (2 Profiles)	2.179
Struts [73]	90K/20K	753 (2 Profiles)	8.447
Findbugs [27]	100K/ 50K	1808 (3 Profiles)	22.267
Spring [72]	500K/90K	7973 (3 Profiles)	50.909
EclipseLink [22]	700K/200K	7117 (3 Profiles)	177.978

Concerning external validity, Jump sets the focus on Java annotations. Many libraries embrace them, and real-world cases provide validity for annotated Java code [66]. However, we cannot claim any results outside of Java.

### Scalability evaluation

**Fig. 14:**
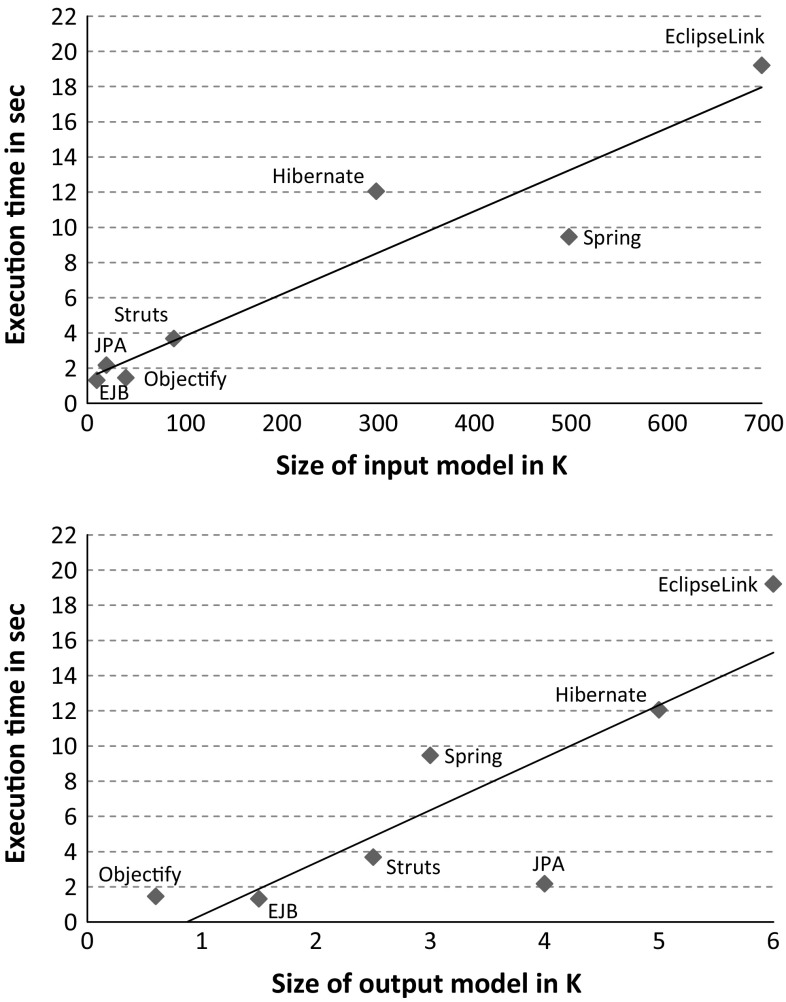
Ratio of model size and execution time for UML profile generation

**Fig. 15:**
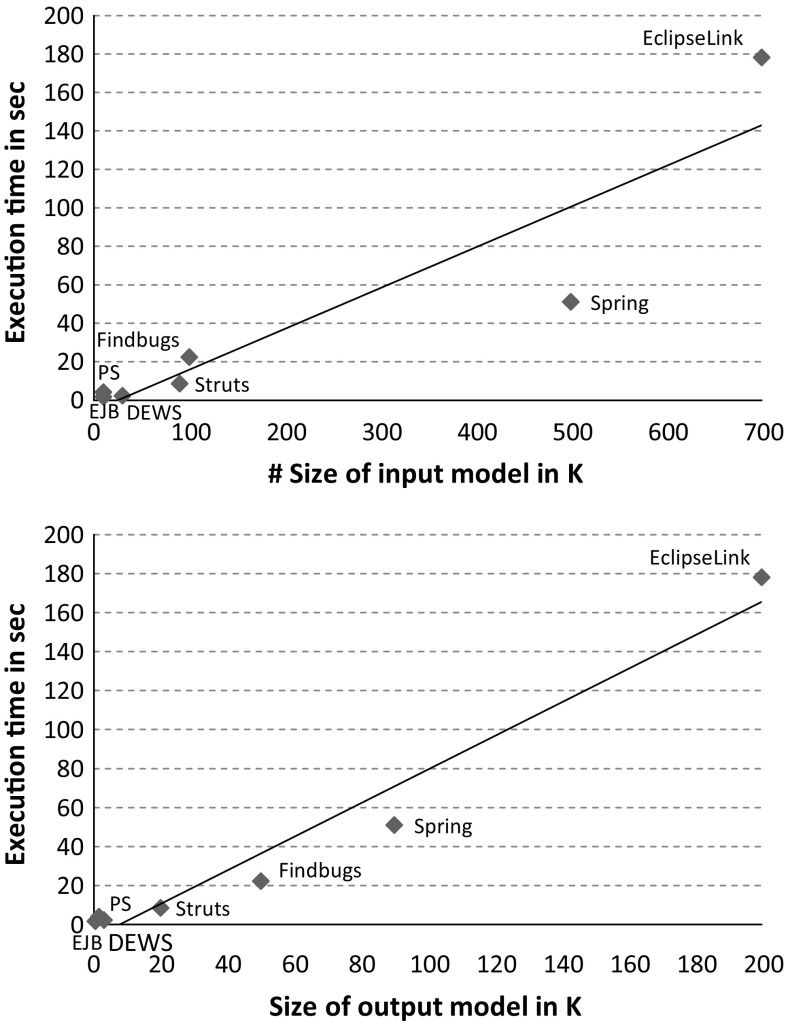
Ratio of model size and execution time for profiled UML model generation

To report on the scalability of the Jump tool, we measured the execution time of applying the *JavaCode2UMLProfile* and *JavaCode2ProfiledUML* transformations to several libraries used in practice and real-world applications. In order to answer (*RQ*3), these chains were executed in Eclipse Luna 4.4.2 with Java 1.8 on commodity hardware: Intel Core i5-2520-M CPU, 2.50 GHz, 8,00 GB RAM, Windows 7 Professional 64 Bit. Tables 5 and 6 summarize our obtained results by emphasizing (1) the number of *elements* in the intermediate Java model, i.e., the input of the transformations, and the produced UML profile / model, i.e., the output of the transformations, (2) the number of *declared* and *applied stereotypes*, and (3) the measured *execution times*. Two results are accompanied with scatter plots, see Figs. 14 and 15, which show the ratio of model size and execution time for UML profile generation and profiled UML model generation, respectively. The scatter plots include a linear regression curve to show the trend of increasing execution time w.r.t. growing model size by considering both input and output models.

The rationale behind our selection of libraries and applications is to consider small-sized to large-sized libraries and applications with varying number of declared and applied stereotypes. Clearly, the size of the input models passed to the transformations as well as the size of the produced output models has a strong impact on the execution time of the Jump tool as they are traversed throughout the generation of profiles and profiled models. Regarding the UML profile generation, the number of generated stereotypes is another main factor that impacts on the execution time. Generally, the more stereotypes are generated, the more extensions to UML meta-classes and features of these stereotypes need to be created. As a result, the number of produced stereotypes has a strong impact on the size of the generated UML profile. For instance, even though the JPA is compared to Objectify smaller in size, the execution time is higher because a lot more transformation rules are applied when considering the number of declared stereotypes. Similarly, the execution time of generating a profile for EclipseLink is twice as high as it is for Spring, which can be explained by the major difference in the number of declared stereotypes, i.e., 127 versus 63.

Regarding the generation of profiled UML models, the more stereotypes from different UML profiles are applied, the higher is the execution time. Similarly, the number of applied stereotypes and their respective profiles influences the execution time. For instance, in the Petstore (PS), stereotypes are applied from 12 different profiles, which explains the higher execution time compared to DEWS core, even though the input model of the latter is larger in size. Considering the measured results for Struts and Findbugs show the strong impact of the size of the generated output model and the number of applied stereotypes on the execution time. Even though the input models of Struts and Findbugs are almost similar in size, the execution time of the latter is more than twice as high as of the former. This can be explained by the fact that the output model and the number of applied stereotypes in the case of Findbugs are double in size compared to Struts. If the size of both input and output models is as large as it is in case of EclipseLink, the high memory consumption and as a result the excessive use of garbage collection may also be an additional factor that influences execution time. This is one reason why the execution time for EclipseLink is above the overall estimated trend, see Fig. 15. Considering the execution time of the profiled UML model generation, it is generally higher compared to profiles because the class structure of the former is much larger in size compared to the latter. For instance, considering EclipseLink and the number of generated stereotypes compared to classes the factor is almost 30.

### Practicability evaluation

As Jump is intended to be used by engineers that produce platform-specific profiles not only to support transformations of a RE process but also in an FE one, we report on our experiences of applying it in the context of a software modernization to the cloud that involves both processes. In doing so, we elaborate on the use case motivated in Sect. 2.1, where a change of the data access platform is discussed and provides insights into the transition of a JPA-based solution to an Objectify-based solution aimed to be hosted on the Google Cloud Platform. This cloud-based solution allows entities to be retrieved not only by plain service classes but also via a REST-based client which basically resembles Google’s Cloud Endpoints service^5^ (cf. e.g., [23]). To carry out the transition toward a cloud-based solution, we apply Kazman’s “horseshoe” [44] in light of model-driven software engineering (MDSE) and cloud-oriented software modernization. As a result of applying advanced MDSE techniques, we reverse-engineered an environment-independent^6^ domain model in a quality that allowed us to directly refine it toward an environment-specific one from which we generated the complete cloud-based solution. In this modernization scenario, we particularly emphasize the benefit of annotation-based modeling to improve the quality of the reverse-engineered domain model and to generate model artifacts that could have hardly been generated otherwise. Based on our insights gained from the outlined modernization scenario, we aim to answer (*RQ*4).

**Fig. 16:**
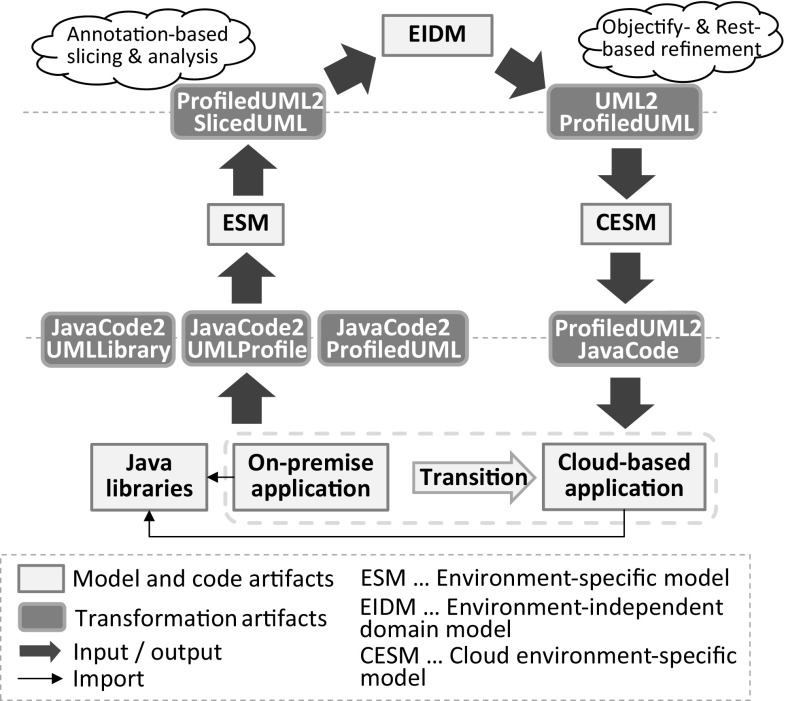
Modernization roadmap to the cloud

#### Jump in action

**Fig. 17:**
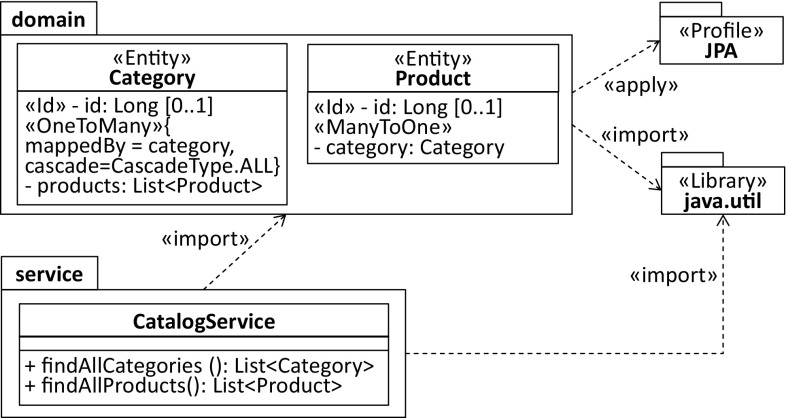
Reverse-engineered model specific to the Java and JPA environment (ESM)

The conceptual overview shown in Fig. 16 relates all the code, model, and transformation artifacts involved in our modernization scenario. Considering the first step in the RE process, a UML model that captures the complete on-premise application from a structural perspective is generated. Moreover, Java libraries are reverse-engineered into corresponding UML libraries and UML profiles as they enable succeeding transformations to exploit information that is specific for the current environment mainly to provide abstractions over the initially generated environment-specific model (ESM) and to improve their quality. For instance, the profiled model represented in Fig. 17 captures the two entities and the service class to retrieve them as shown in Listing 11.
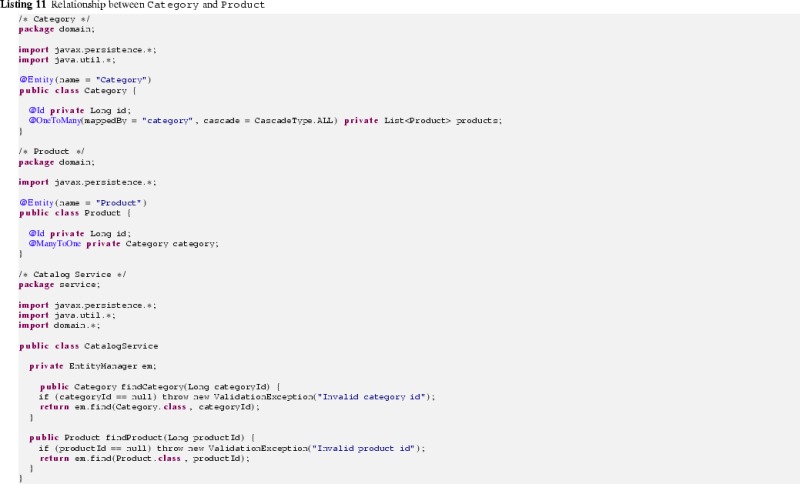


This model is specific to the Java environment as the products property and both methods findAllCategories and findAllProducts are of type java.util.List and the JPA as all the applied stereotypes refer to corresponding annotations of it.

**Fig. 18:**
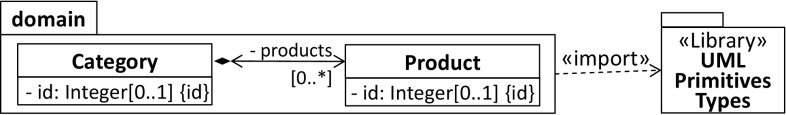
Environment-independent domain model (EIDM)

In the second step of the RE process, the domain model is sliced from the ESM by withdrawing all model elements that do not denote entities and turning Java-specific types into corresponding UML concepts. Considering the former, we have implemented an annotation-based slicer where the slicing criterion that captures the point of interest is a set of stereotypes. Model elements to which at least one of the stereotypes is applied are considered as part of the computed model slice (e.g., Entity and Embeddable). Moreover, stereotypes applied to the ESM are analyzed mainly to improve the quality of the sliced environment-independent domain model (EIDM). The EIDM is shown in Fig. 18.

For instance, the composition relationship between Category and Product of the EIDM has been generated on the basis of the @OneToMany stereotype applied to the products property of the Category contained by the ESM. The selected CascadeType allows the composition relationship to be derived where its member ends are determined by the property to which the @OneToMany stereotype is applied and the property assigned to the mappedBy element. Without this detailed consideration of the @OneToMany stereotype, we would at best be able to generate properties with the respective types, i.e., Category and Product. Concerning Java-specific types, we mainly turned collection types of properties into multivalued properties and primitive types known from Java into primitive types offered by UML.

**Fig. 19:**
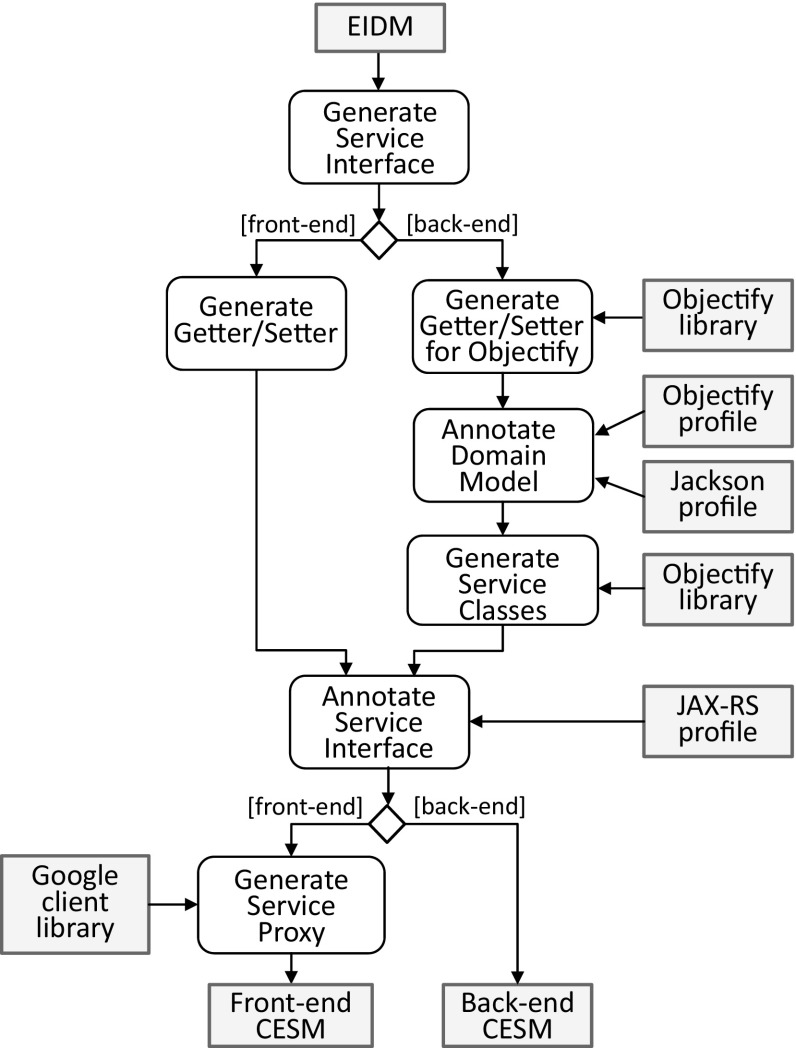
Refinement of EIDM toward CESM

Based on the reverse-engineered EIDM, we started the refinement toward the target environment as part of the FE process. In fact, we produced two different cloud environment-specific models (CESM) one for the “front-end” that is considered as the API used by the REST-based clients and one for the “back-end” that is connected to the cloud datastore of the Google Cloud Platform. Client requests are delegated from the front-end to the back-end. How the corresponding front-end CESM and back-end CESM is generated from the EIDM is depicted in Fig. 19.

**Fig. 20:**
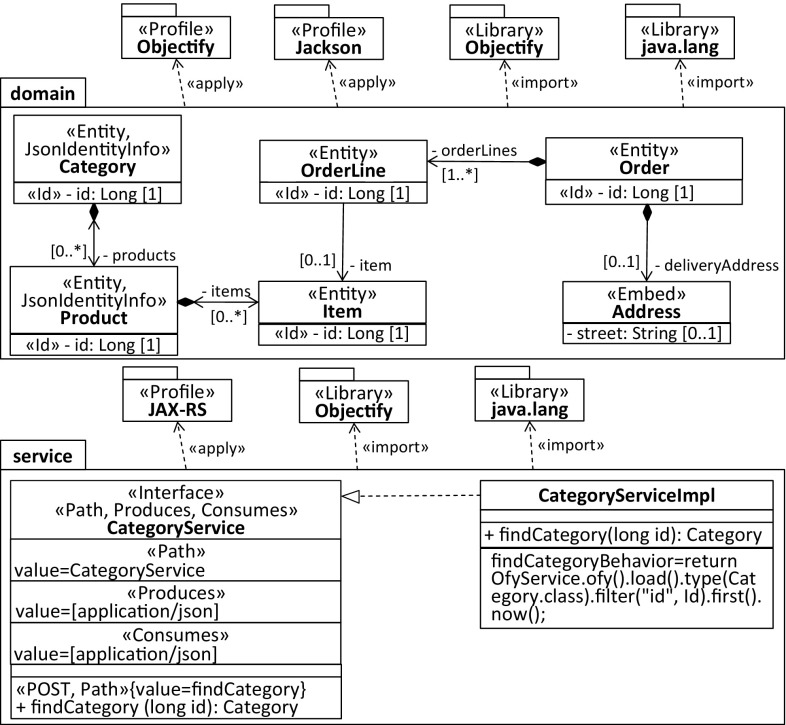
Modernized back-end model specific to the target cloud environment

**Fig. 21:**
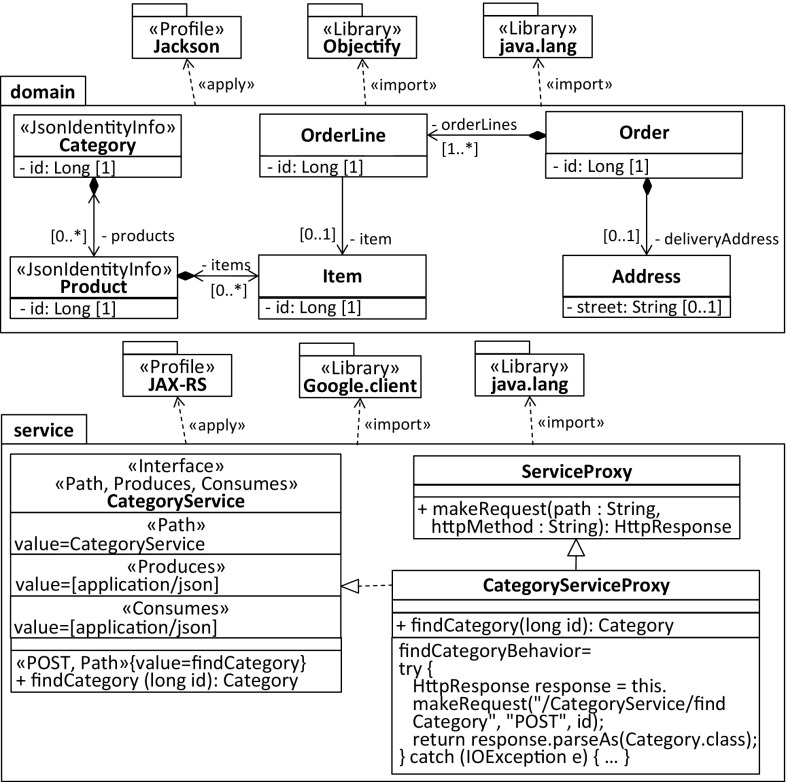
Modernized front-end model specific to the target cloud environment

It shows the process and the main models, profiles, and libraries involved in the refinement. In the first step, service interfaces and service methods are generated for the entities of the domain model to create, read, update, and delete them. For instance, the CategoryService interface in Fig. 20 and 21 is a result of this first step.

Depending on whether the front-end CESM or the back-end CESM is generated, getter/setter methods are generated either in a standard way or specific for Objectify. In case of generating the back-end CESM, the EIDM is annotated with stereotypes of the Objectify profile and the Jackson^7^ [39] profile before concrete service classes for the service interfaces are generated. For instance, an explicit composition relationship between two entities where the contained one cannot be identified by a dedicated property indicates that the latter entity needs to be embedded by the former entity. This embedding of entities can be expressed in Objectify via the Embed stereotype, e.g., the Address domain class in Fig. 20. Moreover, the Id stereotype is required for generating the behavior of service methods where the identifier of an entity needs to be accessed, e.g., the method findCategory(long entityId) of the service class CategoryService in Fig. 20. In cases of cyclic or bidirectional relationships between entities the stereotype, JsonIdentityInfo is required for instructing the serialization and de-serialization process as part of a REST-based solution to turn bidirectional relationships into cross-references of a tree-based structure as determined by JSON.

Annotating the service interfaces with stereotypes of the JAX-RS^8^ [40] profile is a prerequisite for exposing them to clients. Moreover, these stereotypes are required to generate the behavior of the service proxies as part of the front-end CESM. For instance, to correctly deal with the HttpResponse object of the findCategory(long entityId) method in the CategoryServiceProxy, the Path stereotypes applied to the service interfaces and service operations, and the stereotypes determining the REST method need to be accessed.

Having generated both the front-end CESM and the back-end CESM as a result of the above refinement steps, the corresponding Java code can be produced from them. For that reason, we have adapted the Java-based model transformer provided by Obeo Network in several respects.

1. Stereotypes for which the corresponding annotations need to be produced
2. OpaqueBehaviors that are used to generate method bodies
3. ElementImports as they indicate the required import statements of the Java code

Listing 12 shows the generated back-end Java code for the Category domain class, whereas in Listing 13 its front-end Java code is given.
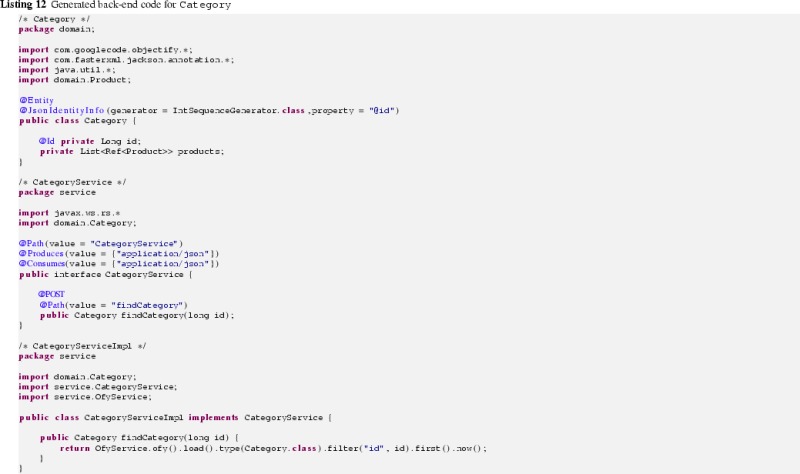
 
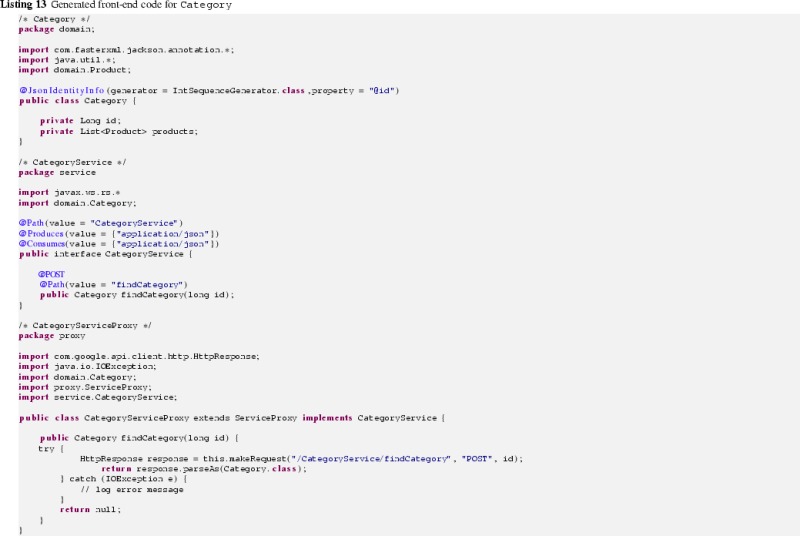


#### Synopsis

We have shown the transition of a non-cloud application into a cloud application with a focus on modernizing the data access layer. As a result of the RE process, we obtained an environment-independent domain model from which we generated environment-specific models for the back-end as well as the front-end of the REST-based solution hosted on the Google Cloud Platform. Objectify is used to manage the access to the cloud datastore. Table 7 gives some quantitative characteristics of the eCommerce web application we dealt with in the modernization scenario.

**Table 7:** Quantitative characteristics

Model element types	Number of model elements					
	ESM	EIDM	CESM			
			Front-end		Back-end	
			Reused	Generated	Reused	Generated
Classes/interfaces/enumerations	39	9	9	13	–	22
Properties	157	49	40	5	–	40
Operations	263	–	85*	74*	–	153*
Annotations	287	–	–	78	–	95

$$^*$$ Including behavior

The ESM reflects its structural elements, whereas the EIDM captures the essence of the entities without operations to access their properties as these operations may not fit the requirements of the target environment anyhow. Finally, the CESM’s produced for the front-end and the back-end represent the structure as well as the behavior of the cloud-based solution. We distinguish model elements that can be reused from model elements that are generated as part of the FE process. In fact, the domain classes of the original implementation can obviously be reused for the front-end. Service classes are newly generated as they need to be appropriately annotated and the service proxies have not existed in the original implementation. Regarding the back-end, all the artifacts are newly generated as the change from JPA to Objectify requires to modify the domain classes and the service classes to access them. Finally, Table 8 summarizes the benefits of annotation-based modeling in the context of our modernization scenario. While the presented stereotypes were automatically applied in our modernization scenario by model transformations, other stereotypes may directly be applied by engineers in a manual refinement step. For instance, Objectify provides annotations to index properties of entities or cache retrieved entity instances. As all the annotations of Objectify are captured by respective stereotypes on the model level, the engineers have full control over such platform-specific decisions at any phase of the forward engineering process.

**Table 8:** Benefits of annotated models

Profile	Stereotype	Benefit
Objectify	$\texttt {Entity}$	Indicates entities that need to be persisted and allows the entity registry to be generated
	$\texttt {Id}$	Indicates properties that identify domain classes and allows the behavior of service classes to be generated
Jackson	$\texttt {JsonIdentityInfo}$	Allows cross-references to be produced
JAX-RS	$\texttt {Path}$	Allows the URL of the service request to be generated
	$\texttt {POST, PUT, DELETE}$	Indicates the employed REST method and allows the service request to be completed

## Related work

With respect to the contribution of this paper, namely to generate UML profiles from Java libraries, we consider three threads of related work. First, we discuss mappings between Java and UML because Jump builds on existing efforts in this respect. Thereafter, generative approaches dealing with UML profiles and Java Annotation Types are discussed. Finally, we consider approaches that support metamodel generation from programming libraries.

### Mapping Java and UML

The elaboration on the mapping between Java and UML has a long tradition in software engineering research [26, 36, 46, 54]. Round-trip engineering for UML and Java has been extensively studied in the development of FUJABA [54]. One particular concept of UML that received much attention in the context of Java code generation is the association concept [2, 33, 34]. However, none of these approaches consider the transformation of annotation types and their applications from Java to UML. The only exception is the mTurnpike approach [76] that considers Java annotations on the model level. Therefore, round-trip transformations between UML models and Java code are realized by considering stereotypes and annotations in the transformations. In contrast, Jump sets the focus on the automated generation of UML profiles that facilitate round-trip transformations or transformations in general. Besides academic efforts, today’s modeling tools support the transformation of Java code to UML models, and vice versa. Their current capabilities and limitations w.r.t. Jump are discussed in Sect. 6.1.

### Generating UML profiles and Java annotation types

The only approaches we are aware of that deal with automated generation of profiles fall into the research area concerned with bridging the gap between MOF-based metamodels and UML’s profile mechanism, which is also related to the discussion of an external domain-specific modeling languages (DSML) compared to an internal DSML where the host language is UML [28]. Considering the latter, they are internal in the sense that they are embedded in a host language [53] providing the base elements for which extensions and constraints are developed. In contrast, external DSMLs are built from scratch and have their own custom concepts without explicit relationships to any existing language. Mernik et al. [53] discuss when and how to develop internal and external DS(M)Ls. Several papers discuss the pros and cons of these approaches, e.g., Selic [71] and their combination, e.g., Weisemöller and Schürr [77].

Visualizing domain-specific models in UML with profiles is discussed in [35]. Abouzahra et al. [1] present an approach for interoperability of UML models and DSML models based on mappings between the DSML metamodel and the UML profile. Brucker and Doser [13] go one step further and propose an approach for extending a DSML metamodel for deriving model transformations able to transform DSML models into UML models that are automatically annotated with stereotypes. A related approach is presented by Wimmer [78], where mappings between the UML metamodel and a DSML metamodel are defined and processed to generate UML profiles for the given DSMLs.

Considering the generation of Java annotation types from DSML models, Ann [18] is a recent approach for modeling Java annotation types. It provides code generation facilities to produce the Java code of modeled annotation types as well as respective annotation processors that implement validation rules for annotations applied to program element. For instance, the Entity annotation type of the JPA requires that one or several attributes of the annotated Java class define the primary key. One possibility to define it is to apply the Id annotation type to an attribute of the respective Java class. Validating invariants can also be achieved for UML models by associating OCL constraints with stereotypes. In this case, the validation would be carried out before the Java code is actually generated from the UML model.

### Generating metamodels

To the best of our knowledge, there is only one automated approach for generating modeling languages from programming libraries. All other automated approaches that deal with exploring libraries, such as [14], set their focus on the generation of domain models rather than a language.

API2MoL [16] deals with generating metamodels based on Ecore [21] from Java APIs as well as models conforming to the generated metamodels for Java objects instantiated from the Java APIs, and vice versa. As a result, an external DSML is generated from a Java API. While the general idea and motivation of the API2MoL approach are comparable to Jump, there is a significant difference on how the DSML is realized. Jump targets UML modelers that are familiar with UML class diagrams and generates internal DSMLs by exploiting UML’s language-inherent extension mechanism, i.e., *UML Profiles*. Furthermore, annotations are not explicitly considered in the metamodel generation process of API2MoL. One possible reason for neglecting them is that standard versions of current meta-modeling languages, such as Ecore, do not support language-inherent extension mechanisms out of the box [50]. Antkiewicz et al. [3] present a methodology for creating framework-specific modeling languages. While we aim for an automated approach, Antkiewicz et al. use a manual one to create the metamodel and the transformations between model instances and instantiated objects of the frameworks. Again, annotations are not captured by the created languages. Finally, Noguera et al. [55] propose the extraction of annotation models in terms of class diagrams from a set of annotation types with the purpose to define validation constraints for the consistent use of annotations. They mention the study of the relationship of stereotypes and annotation types as interesting subject for future work, only.

Research of related fields considers ontologies as a kind of (meta)model [32]. In particular, research on ontology extraction from different artifacts is subsumed under ontology learning [20]. We are aware of only one approach for extracting ontologies from APIs [68]. It neglects, however, annotations. Furthermore, most of the current ontology learning approaches focus on the extraction of concepts and their taxonomic relationships. Finding non-taxonomic relationships (e.g., associations between classes) and intrinsic attributes are the least considered problems [43] in this field.

### Synopsis

To summarize, Jump is—to the best of our knowledge—the first approach to generate standard-compliant UML profiles from Java libraries that exploit annotations. While other existing approaches are capable of producing (meta)models from Java code, the annotation concept has not received much attention. This is, however, in contradiction with the frequent use and ever-growing importance of the annotation concept on the programming level. Therefore, support for annotations on the model level has to be provided. We applied an internal DSML approach by exploiting the language-inherent extension mechanism of UML. It perfectly suits the annotation mechanism of Java. As a result, we close an important gap between programming and modeling.

## Conclusion

With Jump, we proposed an approach to close the gap between programming and modeling concerning annotation mechanisms. We set the focus on the “Java2UML” case and demonstrated the feasibility of Jump by generating high-quality UML profiles for numerous Java libraries used in practice and by applying it to a practically relevant modernization scenario including both RE and FE processes. The results gained by our evaluation are promising, and an extensive set of profiles is already available for leveraging annotation-based modeling.

Still, a number of future challenges remain to further integrate programming and modeling. Some interesting differences between Java annotations and UML profiles remain to be explored. On the UML side, inheritance between stereotypes is possible, a concept that is not supported by Java for annotation types. Thus, the design quality of automatically generated UML profiles can be enhanced by exploiting inheritance.

On the Java side, retention policies determine at which stages annotations are accessible. UML stereotypes are considered only at design time. Therefore, an interesting line of future work is to support stereotype applications also during run time, which becomes especially interesting for executable models, a research area that is currently experiencing its renaissance by the emergence of the fUML standard [62] and work in this context (cf. e.g., [52]).

Regarding the novelties of Java 8, we plan to study how stereotypes can be applied to the use of a type in UML in analogy to type annotations in Java. However, this would require the possibility to annotate not only model elements in UML, but also the references between model elements which is currently not possible with UML profiles. Moreover, we aim to study the support of annotations in other programming languages, e.g., by investigating attributes in C# and decorators in Python, and how these concepts correspond to UML profiles.

In order to allow UML profiles to be applied to a wider range of modeling languages that support class-based representations similar to UML, our idea is to generalize them based on EMF profiles [50]. Finally, as we set the focus in this work to platform-specific profiles, we plan to extend this scope to profiles that capture annotations independent of platforms, thereby shifting their application to a more conceptual level.

## Footnotes

1 The UML specification contains a simplified EJB profile to discuss the benefits of the profile mechanism and demonstrate its application.

2 Several classifiers, relationships, and features are omitted. We restructured some relationships for reasons of comprehensibility [8]. For instance, in the standard UML metamodel Class inherits indirectly from NamedElement; hence, we reduced the intermediate meta-classes forming a deep inheritance hierarchy.

3 A stereotype is applicable to a base element if it is an instance of a meta-class extended by the stereotype. Considering the Entity stereotype, it extends the meta-class Type. As a result, it can be applied to all instances of the meta-class Type.

4 Obeo Network: http://marketplace.eclipse.org/content/uml-java-generator.

5 Google Cloud Endpoints: https://cloud.google.com/appengine/docs/java/endpoints/.

6 The term environment is used in analogy to platform as introduced by the MDA paradigm.

7 Jackson is a JSON processor.

8 JAX-RS is a Java API for RESTful Web Services.
